# Can bone marrow‐derived mesenchymal stem cells change liver volume?: A case report

**DOI:** 10.1002/jgh3.12466

**Published:** 2020-12-17

**Authors:** Kwangmin Kim, In Sik Shin, Hui‐Jae Bang, Sanghyun An, Gaesung Ha, Hyun Soo Kim, Keum Seok Bae

**Affiliations:** ^1^ Department of Surgery Yonsei University Wonju College of Medicine Wonju South Korea; ^2^ Trauma Center Wonju Severance Christian Hospital Wonju South Korea; ^3^ Wonju Severance Surgical Research Group Wonju Severance Christian Hospital Wonju South Korea; ^4^ Pharmicell Co., Ltd. Sungnam South Korea; ^5^ Kim's Stem Cell Clinic Seoul South Korea

**Keywords:** computed tomography, liver cirrhosis, liver volume, mesenchymal stem cell

## Abstract

Several studies have described the effectiveness of mesenchymal stem cell (MSC) transplantation in patients with liver cirrhosis (LC). However, in the majority, biochemical tests, clinical features, and pathologic results were used rather than radiologic tests to compare treatment outcomes. A 57‐year‐old male visited a stem cell clinic with a diagnosis of LC attributed to hepatitis B virus. This patient took tenofovir and diuretics at the initial presentation and was administered bone marrow‐derived MSCs twice via hepatic intra‐arterial infusion. Subsequently, the patient's clinical symptoms and biochemical tests (aspartate aminotransferase, alanine aminotransferase, albumin, total bilirubin, international normalized ratio, creatinine, alpha‐fetoprotein) improved. Computed tomography findings showed loss of ascites, reduced nodularity, and especially increased liver volume, which suggested that MSCs have meaningful effects on liver volume, as well as improving liver function.

## Graphical abstract

Indeed, many studies have reported the effectiveness of mesenchymal stem cell (MSC) transplantation in patients with liver cirrhosis (LC). However, in most of these studies, biochemical tests, clinical features, and pathologic results were used rather than radiologic tests to compare treatment outcomes. This case report demonstrates the effectiveness of MSC infusion based on computed tomography findings of an increase in liver volume in a patient with hepatitis B virus associated LC.

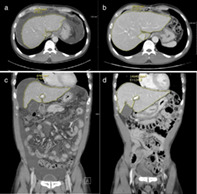

## Introduction

Liver cirrhosis (LC) is the end result of long‐term liver damage, and its associated morbidities impose significant health‐care burdens. Liver transplantation is the only definitive therapeutic option for these patients. However, the paucity of donors, rejection, and the high cost are hindering factors. In addition, lifelong immunosuppression is a worrisome issue. Cell‐based regenerative therapies, particularly adult stem cell‐based therapies, are evolving as viable clinical alternatives.^1^ Indeed, many studies have reported the effectiveness of mesenchymal stem cell (MSC) transplantation in patients with LC.^2^, ^3^, ^4^ However, in most of these studies, biochemical tests, clinical features, and pathologic results were used rather than radiologic tests to compare treatment outcomes. This case report demonstrates the effectiveness of MSC infusion based on computed tomography (CT) findings of an increase in liver volume in a patient with hepatitis B virus (HBV)‐associated LC. The MSCs were obtained from bone marrow and administered via hepatic artery.

## Case report

A 57‐year‐old Korean man presented at a stem cell clinic with a diagnosis of HBV‐associated LC with abdominal distension and peripheral pitting edema. Eight weeks before this visit, the patient was admitted to a local medical center with the same symptoms, and biochemical tests and radiologic findings resulted in a diagnosis of LC. At this presentation, the symptoms had not improved despite the patient being on tenofovir and diuretics (spironolactone, furosemide) for 8 weeks, and the patient had Child‐Pugh (CP) grade B (score 9) disease with gross ascites, spider angioma, umbilical hernia, and peripheral pitting edema but no overt jaundice. The liver was hardly palpated in the epigastric area. Endoscopy showed esophageal varix and CT revealed liver shrinkage, a huge amount of ascites, and splenomegaly.

Following the provision of informed patient consent, MSC transplantation was conducted on two separate occasions. A total of 20–25 milliliters (mean ± SD: 23.1 ± 11.5 mL) of bone marrow (BM) aspirates were obtained under local anesthesia from the posterior iliac crest during the visit before treatment. All manufacturing and product‐testing procedures for the generation of clinical‐grade autologous MSCs were conducted in accordance with good manufacturing practice (Pharmicell Company Limited, Sungnam, Korea). Mononuclear cells were separated from BM by density gradient centrifugation (HISTOPAQUE‐1077; Sigma‐Aldrich, St. Louis, MO, USA) and washed with phosphate‐buffered saline (PBS). Cells were resuspended in Dulbecco's modified Eagle's medium‐low glucose (DMEM; Gibco, Grand Island, NY, USA) containing 10% fetal bovine serum (Gibco) and 100 U/mL penicillin/100 μg/mL streptomycin (Gibco). Cells were then plated at 2–3 × 10^5^ cells/cm^2^ into 75‐cm^2^ flasks and maintained in a humidified 5% CO_2_ atmosphere at 37°C for 5–7 days, after which nonadherent cells were removed by replacing the medium. Cells were then cultured for another 2–3 days, and when near confluent (70–80%), adherent cells were detached using trypsin containing ethylene diamine tetra‐acetic acid (EDTA; Gibco) and then replated at 4–5 × 10^3^ cells/cm^2^ in 175‐cm^2^ flasks. Cells were serially subcultured to passage 4 or 5 for infusion (4.4 ± 0.5 passages [mean ± SD]).

On the day of injection, MSCs were harvested using trypsin/EDTA, washed twice with PBS and once with saline solution, and then resuspended at a final concentration of 5 × 10^6^ cells/mL in 10 mL of saline solution. The clinical criteria used were: viability >80% and the absence of microbial contamination (bacteria, fungus, virus, or mycoplasma) when tested 3–4 days before administration and in the presence of CD73 and CD105 in >90% of cells and of CD14, CD34, and CD45 in less than 3% of cells by flow cytometry. Cells (5 × 10^7^ in 10 mL of saline) were injected via the right hepatic artery using a coaxial angiographic catheter (Boston Scientific, Natick, MA, USA). BM‐MSCs were injected using a coaxial angiographic catheter (Boston Scientific) placed in the hepatic artery. All procedures following the first and second MSC administrations were performed on an outpatient basis. Routine biochemical parameters, symptoms and signs, diuretic requirements, and overall CP score were assessed retrospectively.

After first and second infusions, aspartate aminotransferase (AST), alanine aminotransferase (ALT), albumin, total bilirubin, international normalized ratio (INR), creatinine, alpha fetoprotein, and quantitative HBV DNA polymerase chain reaction (PCR) values were improved compared to the laboratory results at initial presentation. Platelet count fell from 93.1 (1000/μL) at the initial presentation to 78.0 (1000/μL) at the last follow‐up. The CP score improved after the second infusion and was 5 (class A) at week 80 (Table 1).

**Table 1:** Biochemical parameters and Child‐Pugh scores of our liver cirrhosis patient

	Baseline	Week 0	Week 4	Week 12	Week 16	Week 24	week 80
AST (U/L)	83	First injection	55	49	Second injection	38	35
ALT (U/L)	42		35	31		23	25
Albumin (g/dL)	2.56		3.25	3.76		4.27	4.2
Total Bilirubin (mg/dL)	1.96		1.17	1		1.5	1.4
INR	1.38		1.3	1.3		1.14	1.01
Platelet (1000/μL)	93.1		93.9	92.1		95.4	78
Creatinine (mg/dL)	1.23		0.94	0.99		1.02	1
αFP (ng/mL)	212.3		33.9	18.9		16.4	10.93
Quantitative HBV DNA PCR (IU/mL)	390 000		618	380			33.2
CP score	9		7	5		5	5

αFP, alpha fetoprotein; ALT, alanine aminotransferase; AST, aspartate aminotransferase; CP, Child‐Pugh; DNA, deoxyribonucleic acid; HBV, Hepatitis B virus; INR, international normalized ratio; PCR, polymerase chain reaction.

**Figure 1:**
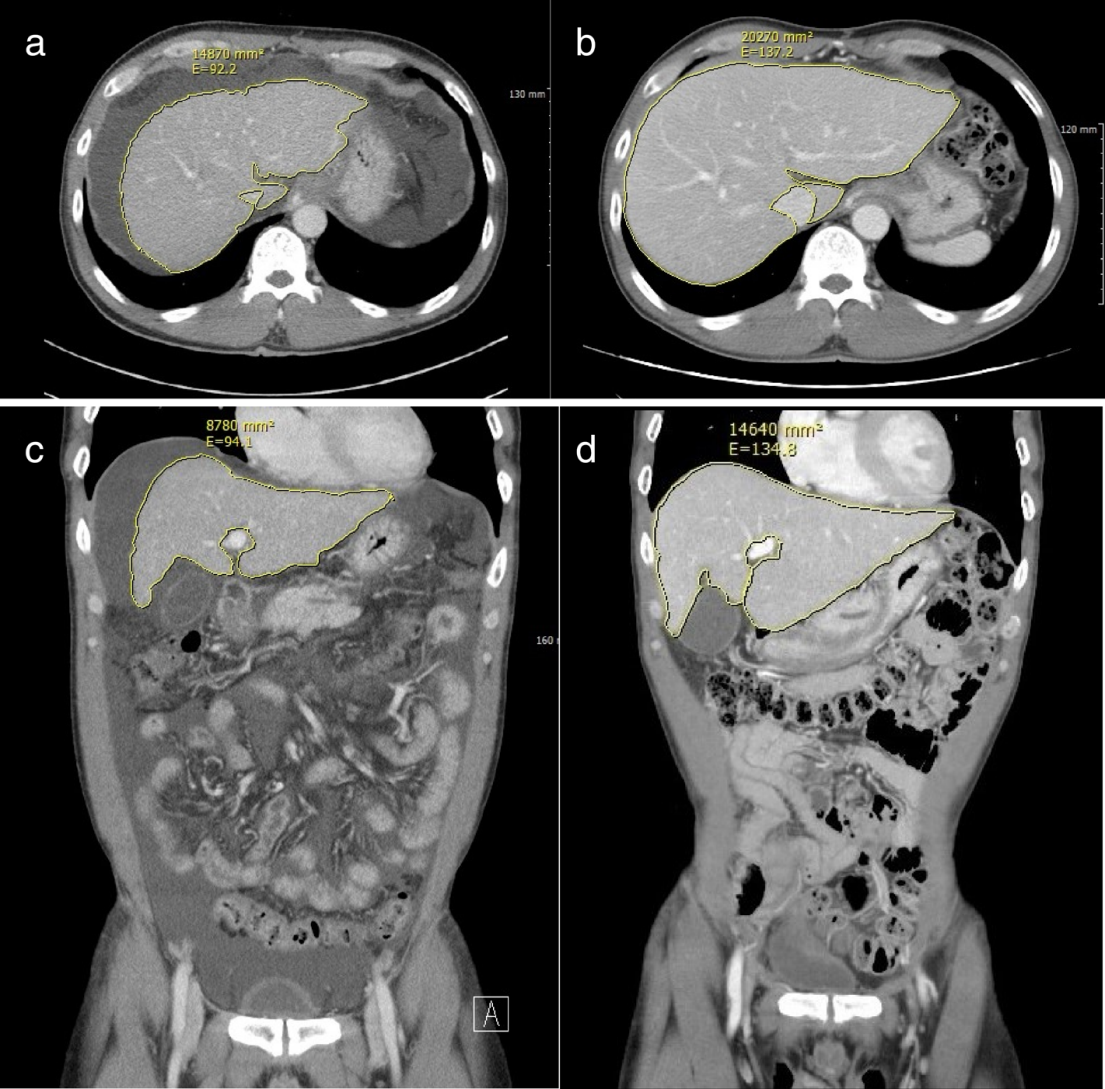
Computed tomography (CT) images taken (a) before mesenchymal stem cell infusion, showing ascites and a liver volume of 14 870 mm^2^, and (b) at 80 weeks after infusion, showing reduced ascites and a liver volume of 20.270 mm^2^. (c) Coronal CT view before treatment showing a huge amount of ascites and a liver volume of 8780 mm^2^. (d) Coronal CT view at 80 weeks after treatment showing improved ascites and a liver volume of 14.640 mm^2^. Images with largest liver volumes were chosen for the CT images.

At 4 weeks after treatment, the amount of ascites decreased as demonstrated by follow‐up ultrasonography, and pitting edema disappeared on both legs. Diuretics were stopped at this point. Ascites was not observed on follow‐up ultrasonography at week 12 after treatment, and at week 80, ascites, pitting edema, and spider angioma were not visualized. CT performed at initial presentation depicted LC, a huge amount of ascites, and a nodular liver surface, but CT at 80 weeks showed the absence of ascites, a smooth liver surface, and a greater liver volume than were observed on initial CT (from 14 870 to 20 270 mm^2^ in cross‐sectional view and from 8780 to 14 640 mm^2^ in coronal view) (Fig. 1).

## Discussion

Several studies have reported positive outcomes for stem cell therapy based on biochemical and pathologic results in LC patients. Most previous studies have not investigated the effect of MSC transplantation on liver volume. However, Zekri *et al*.^5^ used abdominal ultrasonography to measure liver sizes in a control, a single infusion, and a repeated infusion group but found no significant differences between liver sizes in these groups. Furst *et al*.^6^ included liver cancer patients contraindicated for resection because residual liver volumes would have been insufficient to ensure survival and selectively infused autologous BM CD133+ cells into nonoccluded segment II and III portal branches 2–4 h after portal vein branch embolization (I, IV, V–VIII) to stimulate liver regeneration and enable earlier tumor resection. Liver volumes were found to increase much more rapidly in patients who received stem cells, to the extent that cancer resection could be undertaken much sooner (27 days ±11 *vs* 45 days ±21, *P* = 0.6). In our patient, liver volume increased from 14 870 to 20 270 mm^2^ in cross‐sectional view and from 8780 to 14 640 mm^2^ in coronal view at week 80 after completing MSC infusions.

Many authors have suggested that hepatocyte‐like cell differentiation and MSC to hepatocyte differentiation are promising sources of liver regeneration and the main therapeutic mechanisms responsible for the beneficial effects of MSCs in LC patients.^7^ However, Forbes *et al*.^8^ reported that stem cell‐derived hepatocytes were incompletely characterized in most of these studies in terms of “hepatocyte functions” and that they did not demonstrate in vivo functionality at the endogenous hepatocyte level. In addition, these authors commented that, while there are examples of “hepatocyte‐like cells” being produced from nonhepatic adult stem cells, these cells are unlikely to provide a source of new hepatocytes with sufficient functionality to be clinically relevant. Rather, it was suggested that liver hepatocytes offer a more realistic potential source of hepatic regeneration and that various trophic factors secreted by MSCs play key therapeutic roles by influencing the activities of hepatocytes as MSCs are known to express trophic factors that reduce inflammation, apoptosis, and fibrosis of damaged tissues and stimulate angiogenesis and tissue cell regeneration.^7^

Causes of LC also require consideration. Many studies have targeted alcoholic LC, and because alcohol is not permitted in these patients, few factors meaningfully affect study results and introduce bias. However, when studying the therapeutic effects of MSCs in patients with LC of viral origin, patients who do not take antiviral agents are preferred, but these patients are far more difficult to enroll than patients with alcoholic LC. For this reason, the number of studies on LC of viral origin is smaller than studies on alcoholic LC. Terai *et al*.^9^ conducted a study on the effect of stem cells in patients with LC of HBV or hepatitis C virus (HCV) origin. Eight of the nine patients enrolled had LC of viral origin and received antiviral agent**s** and diuretics. The authors reported improvements in serum albumin, CP score, and alpha fetoprotein. Salama *et al*.^2^ conducted a study on the effect of autologous MSC infusion delivered via the peripheral vein in patients with LC of HCV origin. The authors reported improvements in serum albumin, INR, AST, and ALT. In our patient, AST, ALT, albumin, total bilirubin, INR, creatinine, alpha fetoprotein, quantitative HBV DNA PCR results, and CP score were improved, and improvements in clinical features such as ascites and pitting edema were remarkable after two infusions of MSCs.

The major limitation of this case report is that this patient received an antiviral agent concomitantly. However, we consider our experiences meaningful because CT after two administrations of MSCs showed an increase in hepatic volume, reduced nodularity, and loss of ascites. In addition, after two MSC infusions, the clinical symptoms and biochemical test results improved. Our observations suggest that MSCs might meaningfully increase liver volume and improve liver function in a background of LC. The majority of studies undertaken to assess the effectiveness of MSC implantation in LC patients have used biochemical tests and pathologic reports to assess outcomes rather than radiologic findings to assess liver volume. We hope that the present case study encourages the additional use of radiological findings to clarify the effects of MSCs on liver volume.
